# What means civic education in a digitalized world?

**DOI:** 10.3389/fpsyg.2024.1257247

**Published:** 2024-03-11

**Authors:** Josephine B. Schmitt, Jasmin Baake, Sandra Kero

**Affiliations:** Center for Advanced Internet Studies (CAIS) gGmbH, Bochum, Germany

**Keywords:** civic education, civic literacy, informal learning, social media, digital media, self-directed learning

## Abstract

The hope of reaching diverse and large target groups has motivated civic education practitioners to offer their content on social media. The question has therefore long ceased to be whether civic education should take place on the internet, but rather how civic education goals can be implemented digitally to foster civic literacy. At first glance, the possibility of reaching a broad audience in a short time seems tempting. At a second glance, social media reveals several challenges that can impair educational processes. The present paper discusses the following questions: What are the opportunities and pitfalls of civic education in social media? How can we ensure successful civic education in a digitalized world? In our article, we want to provide an interdisciplinary perspective on the topic by drawing among others from the literature in the fields of media psychology, communication studies, and education science. By integrating insights from various disciplines, our paper seeks to enrich the academic dialogue and to promote a nuanced understanding of the evolving dynamics of civic education in the digital realm. With its practical focus, our paper further aims to underscore the applicability of scientific research.

## 1 Introduction

Social media have fundamentally changed the way we get information and form political opinions. The hope of reaching diverse and large audiences has motivated professional civic education practitioners to create content for these platforms—or to use social media content in educational settings. The question is no longer *whether* civic education^1^ should take place on the internet, but rather *how* civic education goals can be achieved digitally (Jantschek, 2021) to foster political knowledge and positive attitudes towards democracy, political self-efficacy, and the willingness to participate in the political process (Milner, 2010)—in short: *civic literacy* (Alscher et al., 2022).

At first glance, the opportunity to reach a wide audience in a short time through social media seems tempting. At a second glance, social media reveal several challenges that can impair educational processes. These include structural aspects such as automated algorithms, which can bring educational offerings close to problematic content (Schmitt et al., 2018b; Zieringer and Rieger, 2023) or hinder social learning (Brady et al., 2023), as well as hate speech underneath educational products, that can negatively influence the perception and evaluation of these products by users (Ernst et al., 2017).

Social media blur the lines between political information, entertainment, and social place. This not only opens up a wide range of opportunities for professional civic education practitioners. It also creates new power structures in communication: new ways to articulate information, demands, and opinions and to participate in the public discourse (Jarren, 2021). So, it is not only professionals who are engaged in civic education on social media. It is now open to everyone! This not only dilutes the idea of professional (institutionalized) civic education which so far has been predominant in the German-speaking world (Weißeno, 2005; Heldt and Lange, 2021). It also makes it more difficult to identify reliable actors as well as high-quality products for users. From the user’s perspective, this may have serious consequences for their trust in civic education content in social media but also for learning processes and the acquisition of civic literacy. This raises the following questions: What are the opportunities and pitfalls of civic education in social media? How can we ensure successful civic education in a digitalized world?

This paper aims to address these questions from a German perspective on civic education. We aim to provide an interdisciplinary perspective by drawing on literature from fields such as media psychology, communication studies, and education to enrich the academic dialogue. With its practical focus, the paper can support researchers’ self-reflection about whether their scientific work is primarily conducted as a self-contained endeavor or whether it has genuine practical relevance. Thereby, it contributes to the development of a transfer-oriented mindset within the scientific community. By providing future perspectives for researchers *and* educational practitioners in the final chapter, we aim to inspire ongoing efforts in this area. This not only promotes a sense of purpose and direction for researchers, but also helps practitioners stay informed about emerging trends and best practices. In addition, we want to encourage collaboration and knowledge sharing between these two critical stakeholders, leading to more effective advancements in the field.

## 2 What is civic education?

Civic education serves as an integral yet multifaceted aspect of education (Sander, 2002), aiming to foster political participation and informed decision-making for active democratic engagement (Cassel and Lo, 1997; Yoldaş, 2015). The precise definition, including disciplines, methods, and conceptual frameworks, varies and depends on historical, political, and institutional factors within each country (Galston, 1989; Cogan, 1999; Yuen, 2016; Heldt and Lange, 2021). Each perception translates into different values, learning goals, and didactical principles. However, as the IEA report on International Civic and Citizenship Education emphasizes, internationally there is widespread consensus about the learning objectives for civic education (Schulz et al., 2018).

In Germany, the current notion of civic education is closely linked to the country’s totalitarian experiences in the early days of its democracy. After 1945, the development of democratic values and attitudes was considered essential to the establishment of a stable democratic state. Therefore, the Federal Agency for Homeland Services as an independent authority subordinate to the German Ministry of the Interior was founded. It was renamed the Federal Agency for Civic Education [Bundeszentrale für politische Bildung (bpb)] in 1963. This agency aims to educate the German people on democratic principles, “establish the democratic ideal” [Bundeszentrale für politische Bildung (bpb), 2012], and discourage any attempts to reinstate a totalitarian regime. Therefore, civic education in Germany refers to “all consciously planned and organized, continuous and targeted measures by educational institutions to equip young people and adults with the prerequisites necessary to participate in political and social life” (translated from German, Massing, n.d.). According to the Beutelsbach Consensus [Bundeszentrale für politische Bildung (bpb), 2011] civic education *must* be guided by three basic principles: (a) the prohibition of indoctrination, (b) the requirement to reflect the diversity of perspectives and interests that a given (political/societal) problem may represent, and (c) to teach people to understand and analyze their political interests, and to influence society in pursuit of those interests.

In comparison to other countries, civic education has a special place in Germany. It is not only an integral part of the curriculum in German schools. The bpb is an independent nationwide institution that promotes civic education for *all* citizens. It is committed to the Basic Law, human rights, and democratic political culture [Bundeszentrale für politische Bildung (bpb), 2003]. Its principles are non-partisanship and academic balance. Adherence to these aspects is regularly monitored by the Scientific Advisory Board and the Board of Trustees, which consists of representatives of all parties in the German Bundestag [Bundeszentrale für politische Bildung (bpb), 2001]. This allows for a certain degree of independence in the design of educational programs and materials. Its work includes extracurricular political education in both analog and digital spaces such as social media, as well as civic education in schools.

The traditional idea of civic education in Germany is strongly institutionalized and characterized by high standards and expectations of quality as shown above. However, the emergence of a digital landscape, particularly the widespread adoption of as social media as central information source (Newman et al., 2022) challenges these established norms and standards. With the accessibility and ease of content production on social media, virtually *anyone* can engage in providing content that could be perceived as civic education, even if it may not align with the official perspective of bpb or similar institutions. This diversity increases the variety of potential communicators and formats of civic education content enormously—but also impairs content quality. Moreover, the normative ideal of civic education, advocating value-free, unbiased, and objective content [Bundeszentrale für politische Bildung (bpb), 2011; Bundesministerium des Innern und für Heimat, n.d.], is inferior to emotionalizing and polarizing content in terms of social media dissemination and user acceptance (Stieglitz and Dang-Xuan, 2013; Bossetta and Schmøkel, 2023). These aspects pose various challenges to the formation of civic literacy.

## 3 What is civic literacy?

Civic literacy is the main goal of civic education. A basic understanding of civic literacy includes (1) skills and willingness to communicate and cooperate with other people as well as (2) knowledge and skills to make informed decisions about relevant aspects of peoples’ social environment (Van Helvoort, 2019). Alscher et al. (2022) provide a more detailed definition of civic literacy as an interplay of (a) political knowledge about structures, processes, content, and values, (b) political attitudes regarding democracy and pluralistic society, (c) motivation including political interest and internal political efficacy, and (d) the willingness to participate politically and in civil society activities. Various authors use the term *political* literacy. While this term entails a rather narrow understanding of politics—focusing on knowledge of party differences or basic political concepts (Cassel and Lo, 1997; Alscher et al., 2022)—civic literacy goes beyond the strictly political realm and includes a more holistic perspective. This means that social contexts are taken into account whose primary purpose is not political, but in which the political only emerges situationally (e.g., peer group, school).

Structured pedagogical settings and teaching professionals have been considered to be especially important in the formation of civic literacy (Dassonneville et al., 2012; Castillo et al., 2015; Heldt and Lange, 2021). However, many conventional educational practices are criticized for emphasizing limited versions of civic literacy. These practices seem to undermine the experiences of marginalized people, perpetuate inequality, and suppress the creativity necessary to foster transformative and progressive developments (Garcia and Mirra, 2021). In contrast, as “new literacy practices” (Garcia and Mirra, 2021) social media provide an *informal learning space* for fostering civic literacy (on the differences between formal and informal education see, e.g., Eshach, 2007; Rohlfs, 2011).

## 4 Social media as informal learning space

Young people value social media platforms for informal, self-directed learning outside of formalized educational settings (e.g., schools; Rat für Kulturelle Bildung, 2019)—which is a big advantage for the acquisition of civic literacy. Self-directed learning is characterized by the ability of learners to describe their needs and goals without the help of others, define the resources (e.g., support, materials) needed to achieve them, develop, and implement learning strategies based on those resources, and finally evaluate them in terms of learning success (Hiemstra, 1994; Garrison, 1997). Control over the motivation, need, timing, methods, and success of learning lies entirely with the learners (e.g., Wang and Chen, 2020; Zhu et al., 2020). Simultaneously, self-directed learning allows for the selection of content according to people’s needs and interests in terms of content and style. These are high demands on learners, which can have a positive impact on their sense of self-efficacy and learning outcomes (Toh and Kirschner, 2020; Lasfeto and Ulfa, 2023). For civic education practitioners, this means creating content that is truly relevant to their target audiences to ensure successful learning.

Social media further provide a valuable context for self-directed *experiential* learning (for more details see Kolb, 2014)—or as John Dewey put it “learning by doing”—meaning that a broad set of ideas about political processes can be directly learned, and reformed in social interactions within social media platforms. Observing and experiencing how other people negotiate political issues and engage politically can aid in understanding how the political system works and how decision-making processes unfold. Moreover, it can show the importance of being knowledgeable about policies and political candidates while at the same time participating in the process. This is particularly valuable as compared to traditional forms of civic education, social media give marginalized groups a voice (e.g., Fudge and Skipworth, 2017; Jaramillo-Dent et al., 2022). Findings from the UK, for example, show that actors with a migration history serve as empowering figures of identification in a media landscape that is still quite white (Sobande, 2017; Sobande et al., 2020). Studies about LGTBQIA+ influencers show similar results (Li, 2022). Nevertheless, experiential learning has also been proven to be especially successful in structured educational settings (see, e.g., Phillips-Wren and Adya, 2020), it is moreover highly dependent on the (social) context (e.g., quality of political discussions).

While the conditions of the learning environment, and the selection of materials can be controlled by teachers in a pedagogically framed setting, these aspects of civic education are usually left to the users in social media or cannot be controlled at all (e.g., the algorithmic interconnectedness, Schmitt et al., 2018b; Zieringer and Rieger, 2023).

### 4.1 Challenges of self-directed content selection

The diverse social media landscape holds the promise of self-directed “learning pleasure and educational happiness” (translated from German, Burow, 2017), offering new avenues for individual potential. However, a significant challenge for users lies in the competent selection of relevant content. Personal preferences, biases, the desire for confirmation of existing beliefs, and social influence often shape media selection (Sude and Knobloch-Westerwick, 2022). Users may further succumb to clickbait tactics, prioritizing sensationalism, gossip, and short-term engagement over substantive information (Lischka and Garz, 2021). In contrast to professional civic education content, which aims for objectivity, accuracy, reliability, and thorough research, clickbait content tends to attract attention rapidly through sensational or even misleading headlines. This strategy is frequently employed by right-leaning media outlets or extremist actors, lacking ethical commitment to balanced reporting or content creator responsibility (Kaiser and Rauchfleisch, 2020; Rau et al., 2022).

The implications become more pronounced due to the curation algorithms of many platforms, focused on generating high traffic, views, and engagement. Algorithms not only impact information curation but also influence *social* learning (Bandura, 1986), a crucial aspect of civic education. By determining which political topics or opinions are promoted, how topics are interconnected, and how we perceive friends, social media algorithms shape our worldview and influence individual actions, with or without explicit rewards for certain behaviors. Brady et al. (2023) suggest that algorithms leverage social learning *biases*, emphasizing prestigious, in-group, moral, and emotional (PRIME) information—and not content that positively impacts civic literacy. Users, in turn, learn to create more of such problematic content, saturating the digital environment, particularly in morality and politics, with PRIME information. This can lead to social misunderstandings, fueling conflict and spreading misinformation rather than fostering cooperation and collective problem-solving.

The vast and complex content of the social web places high demands on learners, including attention, motivation, media literacy and decision-making skills. Lacking appropriate skills can lead to feelings of overload, stress (e.g., Bawden and Robinson, 2009; Schmitt et al., 2018a), learning refusal (Burow, 2017), and emotional exhaustion (e.g., Sheng et al., 2023). Information overload can be a significant stressor in decision-making, alongside time pressure, complexity, and uncertainty (Phillips-Wren and Adya, 2020). The tendency to select attitude-consistent information further underscores the importance of skillful (political) information navigation.

The outlined challenges of social media emphasize the necessity of media-savvy educators to support self-directed, informal learning processes. They play a crucial role in imparting essential media skills for navigating platforms, finding appropriate content, and grasping structural conditions. They can develop tailored learning opportunities to meet individual learner needs, supporting self-directed learning (Burow, 2017). Their responsibilities further extend to providing constructive feedback and fostering content discussions (Waldron, 2013).

### 4.2 Impact of content and platform characteristics

Research has found positive effects of social media on all facets of civic literacy defined by Alscher et al. (2022): knowledge, attitudes, motivations, and civic engagement (Schmitt, 2016; Oeldorf-Hirsch, 2018; Gagrčin et al., 2022; Gil de Zúñiga et al., 2022; Lee and Xenos, 2022). However, Vraga et al. (2016) showed that political content receives significantly less attention than social content or news. The attention paid to political information on social media has consequences, as inattentive users are more likely to spread disinformation (Pennycook et al., 2021; Pennycook and Rand, 2021). Conversely, those who pay more attention to political information show a weaker relationship between cynicism and political apathy (Yamamoto et al., 2017) and acquire more political knowledge (Eveland, 2004; Eveland and Schmitt, 2015).

It has further been shown, that the style of a media product is important for directing attention: multimodal content (e.g., consisting of images and text) increases attention (Vraga et al., 2016). However, psychological approaches to information processing such as the *Limited Capacity Model of Mediated Message Processing* (Lang, 2000), the *Cognitive Load Theory* (Sweller, 2011), and the *Capacity Model of Comprehension of Educational Content on Television* (Fisch, 2000) assume increased demands on the cognitive reception and processing capacities of users through multimodal content. In contrast to reading texts, multimodal content requires more cognitive resources in the working memory for information processing. However, the more mental effort is invested by the recipient, the deeper the understanding of the content (Salomon, 1983)—resulting, for instance, in higher knowledge acquisition.

Regarding the reception of audiovisual content, for example on YouTube or TikTok, both visual and auditory information must be processed. In the case of educational content users often must process the narrative and educational content and their relationship to each other. The success of information processing depends on user characteristics (e.g., prior knowledge, interest), characteristics of the media product (e.g., complexity of the presentation, temporal organization of the content) and the usage situation (Fisch, 2000).

However, as social media content is embedded in the larger context of a platform, the structural conditions on the platform pose additional challenges for information processing as they increase the complexity of the information (e.g., further links, recommendations, and comments). Unfortunately, providers of educational content on social media have little control over these aspects. Attention can only be directed to a certain extent by selected visual and auditory triggers in a product (Schmidt-Borcherding, 2021).

## 5 Future perspectives

It becomes clear, that Germany’s traditional approach to civic education, characterized by principles such as non-partisanship and academic balance, faces a significant challenge with the increasing digitalization of the public and political sphere. The informal learning space offered by social media is promising, but it is also fraught with difficulties related to the self-directed selection of content and the potential effects of content and platform characteristics. An overarching challenge is the selection and production of trustworthy and valuable content for civic education, highlighting the need for knowledge about potential creators and quality criteria. So, how can we ensure successful civic education in a digitalized world?

It would be helpful to develop a mapping of key actors. This could be used to inform about the differences between actors, the nature of their institutional involvement, their expertise, and the potential quality of their social media content. Additionally, there is a need to develop and evaluate specific criteria for the quality of social media content in civic education that meaningfully complement the principles of the Beutelsbach Consensus [Bundeszentrale für politische Bildung (bpb), 2011].

Providing a comprehensive framework for the evaluation of actors and content in social media would also address the call for more systematic evaluation of effectiveness and more evidence-based development of social media content aimed at promoting civic literacy, bridging the gap between academic research and practical implementation. In this context, we want to emphasize the need for inter- and transdisciplinary approaches in future research. The value of interdisciplinary and transdisciplinary research cannot be overstated when exploring the intricate dynamics of complex phenomena such as user behavior in social media and platform characteristic. Drawing from diverse disciplines allows researchers to employ a range of methods and perspectives to comprehensively examine the multifaceted relationships within social media platforms. This allows for a more holistic understanding of the factors that influence user engagement, network structures, and the impact of digital content on civic literacy. By bridging disciplinary boundaries, not only within but also across the sciences, researchers can shed light on nuanced patterns, contribute to a richer, more nuanced understanding of the complexities inherent in the digital landscape and can foster the relevance of scientific findings for society (Crow, 2010).

We further want to highlight de claim: civic education *with* digital media, not solely *in* digital media (Ernst and Schmitt, 2020). Social media should be viewed as an informal learning practice alongside more formalized approaches, emphasizing the need for conceptual and structural innovation in civic education. Adequate resources, including community management, financial support, and training for educators, are deemed essential for adapting to the current and future media usage habits of diverse target groups in the digital space and to ensure successful learning.

In conclusion, we advocate for a holistic approach to civic education in the digital age that recognizes the intertwined challenges and opportunities of social media, while emphasizing the need for continuous innovation and adaptation to effectively engage learners in the digital realm.

## Author contributions

JS: Conceptualization, Project administration, Supervision, Writing – original draft, Writing – review & editing. JB: Conceptualization, Writing – original draft, Writing – review & editing. SK: Writing – original draft, Writing – review & editing.

## Footnotes

^1^We understand the English term “citizenship education” and civic education synonymously.
